# Pregnancy Outcomes in Women Diagnosed With Attention‐Deficit/Hyperactivity Disorder: A Population‐Based Register Study

**DOI:** 10.1111/acps.70039

**Published:** 2025-10-01

**Authors:** Anneli Andersson, Miguel Garcia‐Argibay, Sofi Oskarsson, Jonas F. Ludvigsson, Paul Lichtenstein, Brian M. D'Onofrio, Catherine Tuvblad, Laura Ghirardi, Henrik Larsson

**Affiliations:** ^1^ School of Behavioural, Social and Legal Sciences Örebro University Örebro Sweden; ^2^ School of Medical Sciences, Faculty of Medicine and Health Örebro University Örebro Sweden; ^3^ Department of Medical Epidemiology and Biostatistics Karolinska Institute Stockholm Sweden; ^4^ Centre for Innovation in Mental Health, School of Psychology, Faculty of Environmental and Life Sciences University of Southampton Southampton UK; ^5^ Department of Medicine Columbia University College of Physicians and Surgeons New York New York USA; ^6^ Department of Paediatrics Örebro University Hospital Örebro Sweden; ^7^ Department of Psychological and Brain Sciences Indiana University Bloomington Indiana USA; ^8^ Department of Psychology University of Southern California Los Angeles California USA; ^9^ IQVIA Solutions Italy s.r.l. Modena Italy

**Keywords:** ADHD, comorbidity, mental disorders, pregnancy outcomes

## Abstract

**Background:**

Maternal attention‐deficit/hyperactivity disorder (ADHD) has been associated with various pregnancy outcomes, but the degree to which that association is explained by concomitant mental disorders and smoking during pregnancy remains unclear.

**Objective:**

To investigate the association between maternal ADHD and pregnancy outcomes.

**Methods:**

Through the Swedish Medical Birth Register, we identified 977,266 women who gave birth to a live singleton between January 1, 2006, and December 1, 2020 (1,617,121 pregnancies). Of these, 1.3% (12,553 women; 17,434 pregnancies) had an ADHD diagnosis prior to pregnancy. The primary outcome was preterm birth (< 37 weeks), with secondary outcomes being postterm birth (> 41 weeks), small for gestational age, large for gestational age, birth weight (≤ 2500, 2501–3500, > 4500 g), acute and planned cesarean section, assisted vaginal delivery, preeclampsia, and gestational diabetes. Generalized linear mixed‐effects models adjusted for maternal age, year of childbirth, maternal education, comorbid mental disorders, and smoking during pregnancy.

**Results:**

There were 1089 (6.6%) preterm births among women with ADHD, and 73,423 (4.9%) preterm births among women without an ADHD diagnosis, corresponding to a crude OR of 1.33 (95% CI 1.25, 1.42). This association attenuated to nonsignificance after adjusting for maternal age, year of childbirth, maternal education, and comorbid mental disorders (adjOR = 1.06, 95% CI: 0.99, 1.13). Fully adjusted models revealed that ADHD was associated with an increased risk of having a large for gestational age baby (adjOR = 1.16, 95% CI: 1.06, 1.26) and undergoing a planned caesarean section (adjOR = 1.16, 95% CI: 1.06, 1.26). Sensitivity analyses using a broader ADHD definition suggested associations with preterm birth (adjOR = 1.09, 95% CI: 1.04, 1.15) and acute caesarean section (adjOR = 1.09, 95% CI: 1.04, 1.13).

**Conclusions:**

After adjustments for comorbid mental disorders and smoking during pregnancy, maternal ADHD was not associated with preterm birth. An increased risk of delivering large for gestational age babies and undergoing planned caesarean sections was found in women with ADHD.

**Summary**

- Significant outcomes
  ○ After adjusting for comorbid mental disorders and smoking, maternal ADHD was not associated with an increased risk of preterm birth.
  ○ Maternal ADHD was associated with a slightly increased risk of delivering a large‐for‐gestational‐age baby.
  ○ Women with ADHD were more likely to undergo planned caesarean sections, even after adjusting for key covariates.
- Limitations.
  ○ ADHD diagnosis and comorbidities were based on specialist care data, possibly missing undiagnosed or primary care cases.
  ○ The study did not account for all potential confounding variables, such as ADHD severity.
  ○ Findings may not be generalizable outside of Sweden due to differences in healthcare systems and ADHD diagnostic practices.

## 1 Introduction

Attention‐Deficit/Hyperactivity Disorder (ADHD) is a neurodevelopmental disorder typically diagnosed in childhood [1]. ADHD can be a lifelong condition, and the diagnosis and treatment of ADHD in adults have increased significantly over time [2, 3]. A recent systematic review on ADHD during pregnancy reported an increased risk of caesarean section, preeclampsia, and infection. Additionally, children born to mothers with ADHD were more likely to be born preterm and require treatment in neonatal intensive care units [4]. In a population‐based study, women with ADHD were found to have an elevated risk of pregnancy outcomes, such as caesarean section, neonatal resuscitation, and decreased rates of spontaneous labor [5]. Notably, these risks were more pronounced in women who started ADHD medication only after delivery, suggesting that the association between ADHD and pregnancy complications may be driven by the diagnosis itself rather than medication use. Similarly, a more recent study found higher rates of pregnancy outcomes, such as preeclampsia, gestational diabetes, and preterm delivery in women with ADHD [6]. An elevated risk of preterm birth among women with ADHD has been reported, and the elevated risk remained also after accounting for covariates such as substance use and comorbid mental disorders [7]. It is important to note that this study defined ADHD based on having a prescription of ADHD medication before, during, or after pregnancy rather than a clinical diagnosis, and used data from 2005 to 2014. As ADHD diagnoses and stimulant prescriptions among women have increased markedly in recent years, a trend that has raised questions regarding diagnostic practices and treatment thresholds [8], our study builds on this work by using clinical diagnoses of ADHD prior to conception and more recent data, offering additional insights into the association between ADHD and pregnancy outcomes.

Individuals with ADHD frequently experience comorbid mental disorders, including depression, anxiety, and bipolar disorder [9], which are independently associated with pregnancy outcomes such as preterm birth, instrumental delivery, miscarriage, and perinatal death [10, 11]. Additionally, adverse health behaviors, such as smoking during pregnancy [12] are more common among individuals with ADHD and are well‐known contributors to poor pregnancy outcomes, such as low birthweight and preterm birth [13, 14]. Despite this, prior studies have often failed to account for these important factors (such as comorbid mental disorders [5, 6]), limiting our understanding of the association between maternal ADHD and pregnancy outcomes, as associations found previously could be influenced by the presence of co‐occurring mental disorders or smoking during pregnancy [15]. Considering these gaps, the objective of this study was to investigate the association between maternal ADHD and pregnancy outcomes while adjusting for key covariates, such as comorbid mental disorders and smoking during pregnancy. By addressing these factors, the present study aims to provide a more comprehensive understanding of the complex interplay between ADHD and pregnancy outcomes. Given the increased attention and diagnostic activity around adult ADHD, especially in women, this study also evaluated whether findings were consistent across alternative definitions of ADHD derived from clinical diagnoses and medication use.

### 1.1 Aims of the Study

This study aimed to provide a nationwide assessment of pregnancy outcomes among women diagnosed with ADHD in Sweden. It examined risks of adverse outcomes, including preterm birth, deviations in fetal growth, low and high birthweight, and obstetric complications. By evaluating the role of comorbid psychiatric disorders and smoking during pregnancy, the study aimed to clarify potential explanatory factors and inform clinical care for this population.

## 2 Methods

### 2.1 Data Sources

We utilized the personal identity number, assigned to permanent residents of Sweden, to link various population‐based registers [16]. The Swedish Medical Birth Registry (MBR), covering approximately 98% of births in Sweden since 1973, provided information on pregnancy outcomes [17]. Information regarding ADHD and comorbid mental disorders was gathered from the National Patient Register (NPR) [18] which contains complete information on diagnoses based on the Swedish versions of the International Classification of Diseases (ICD), from inpatient care since 1987 and specialist outpatient care from 2001. The Prescribed Drug Register (PDR) [19] established in 2005, was used to retrieve information on dispensed medications used in the treatment of ADHD. Maternal education was defined using the Longitudinal Integration Database for Health Insurance and Labour Market (LISA) [20].

### 2.2 Study Population

We identified all women (*n* = 977,266) aged 15 and older who gave birth to live singletons between January 1, 2006 and December 1, 2020 using the MBR, resulting in a total of 1,617,121 pregnancies. Pregnancies ending in stillbirth were excluded due to limitations in the completeness of the stillbirth variable at the time of data extraction.

### 2.3 Exposure

Women were defined as having ADHD if they had received a diagnosis of ADHD in the NPR before conception (ICD‐9: 314 or ICD‐10: F90) or had been dispensed a drug used almost exclusively in the treatment of ADHD (e.g., Anatomical Therapeutic Chemical [ATC] codes: methylphenidate [N06BA04], amphetamine [N06BA01], dexamphetamine [N06BA02], atomoxetine [N06BA09], or lisdexamphetamine [NO6BA12]), in the PDR. This definition has previously been shown to highly correlate with ADHD symptoms (Cohen's *d* = 1.74) [21]. Out of the 977,266 women in the cohort, 12,553 (1.3%) women were defined as having ADHD *prior* to pregnancy. These 12,553 women had in total 17,434 pregnancies. In this study, ADHD prior to pregnancy was defined as a period of at least 1 year between the date of the first ADHD diagnosis or medication usage and the date of giving birth.

### 2.4 Outcomes

From the MBR, we retrieved information on gestational age (weeks), small for gestational age (SGA), large for gestational age (LGA), birth weight, caesarean section (planned and acute), assisted vaginal delivery, and maternal complications (preeclampsia and gestational diabetes). For the included outcomes, a high validity has previously been demonstrated [22].

In line with previous research, gestational age was categorized into preterm birth (< 37 [23, 24]), and postterm birth (> 41 weeks), term birth (≥ 37 and ≤ 41 weeks) being used as the reference category [25]. Small and LGA were defined according to the Swedish fetal growth curve [26], as having a birth weight two standard deviations below or above the mean birth weight for their gestational age, respectively. Normal gestational age was used as the reference category. Birth weight was analyzed as an ordinal variable divided into four categories [27]; ≤ 2500 g (low birthweight), 2501–3500 g (moderate birthweight), 3501–4500 g (reference category), and > 4500 g (high birthweight). Obstetric delivery was divided into unassisted vaginal delivery (reference category), planned and acute caesarean section, and assisted vaginal delivery (forceps or vacuum). Maternal preeclampsia and gestational diabetes were defined as having ICD‐10 codes O14‐15 and O24, respectively.

### 2.5 Covariates

*Maternal age at childbirth*: Maternal age was divided into three categories: 15–24, 25–34, and ≥ 35 years, retrieved from the MBR. *Year of childbirth*: We included the year of childbirth, as there might be secular changes in both the exposure and outcome variables between 2006 and 2020. *Education*: We used data from the Longitudinal Integration Database for Health Insurance and Labour Market (LISA) [20] to define a proxy measure of socioeconomic status as *the highest achieved maternal education at the time of childbirth*, categorized into three educational levels: up to ≤, 10–12, and ≥ 13 years, which refers to compulsory, upper secondary, and postsecondary, respectively. *Comorbid mental disorders prior to pregnancy*: The NPR was used to collect information on co‐occurring mental disorders prior to pregnancy (i.e., depression, anxiety, bipolar disorder, and substance use disorders [SUD]), that have been associated with pregnancy outcomes in previous research [10, 11, 28]. For transparent and complete reporting of covariates [29] all ICD codes used are presented in Table S4. *Smoking during pregnancy*: Smoking during pregnancy has previously been associated with both ADHD [12] and pregnancy outcomes [14, 15]. Information on smoking during pregnancy was obtained from the MBR. Smoking habits were assessed twice by the midwives: early in pregnancy (at the first antenatal visit) and late in pregnancy (Weeks 30–32). We dichotomized and combined smoking habits from early and late in pregnancy by defining “not smoke” as “not smoking” (0) and “1–9 cigarettes/day” and “more than 9 cigarettes/day” as “smoking” (1).

### 2.6 Statistical Analysis

Data management and descriptive analyses were performed with SAS software version 9.4 (SAS Institute Inc., Cary, NC), R 4.0.5 and R Studio [30]. Generalized linear mixed‐effects models were fitted using the lme4‐package to deal with the nonindependence of observations (some women giving birth more than once between 2006 and 2013) and to estimate Odds Ratios (ORs) and 95% confidence intervals. To address the issue of multiple comparisons, we applied the Benjamini‐Hochberg procedure to control the false discovery rate (FDR) [31]. This ensures that all reported statistically significant results are likely to be valid and not false positives. Specifically, the procedure was applied to the *p* values from the fully adjusted models across all pregnancy outcomes included in the main analyses. We evaluated whether the association remained significant after FDR correction using a threshold of q < 0.05. Confidence intervals were not adjusted.

As a first step, we estimated the prevalence and risk of pregnancy outcomes in women with and without ADHD. Second, we adjusted for maternal age, calendar year of childbirth, and maternal education. Third, we further adjusted for comorbid mental disorders prior to pregnancy, including depression, anxiety, bipolar disorder, and SUD. Fourth, we also adjusted for smoking during pregnancy.

### 2.7 Sensitivity Analyses

First, we used a broader definition of ADHD. Women were defined as having ADHD if they had *ever* received a diagnosis of ADHD or had *ever* been dispensed a drug used in the treatment of ADHD (including diagnoses and prescriptions prior, during, and after pregnancy). This was justified based on ADHD being a neurodevelopmental disorder, with a childhood onset [32]. This broader definition yielded a total of 48,653 women being defined as having ADHD. Second, pregnancies exposed to ADHD medication were excluded (*N* = 346 pregnancies). This exclusion was conducted to examine whether the pregnancy outcomes in women diagnosed with ADHD were influenced by medication use during pregnancy. Third, we estimated the association between ADHD and pregnancy outcomes restricted to women giving birth for the first time (*N* = 707,694). This restriction was applied to eliminate potential biases arising from interdependence between pregnancies, as previous experiences could influence subsequent pregnancy outcomes. Fourth, we used a definition of ADHD to be based on either a diagnosis only (*n* = 4290) and medication only (*n* = 4410), prior to pregnancy. This analysis was conducted to address concerns regarding diagnostic accuracy and potential heterogeneity in clinical presentation. We compared the fully adjusted estimates (Model c) for each group and statistically assessed differences in risk using Wald tests. Fifth, we explored the role of comorbid mental disorders (i.e., depression, anxiety, bipolar disorder, and SUDs) by stratifying the sample into three mutually exclusive groups based on the number of co‐occurring mental disorders: no comorbidity (*n* = 1,456,290), one comorbid mental disorder (*n* = 115,622), and two or more comorbid mental disorders (*n* = 45,209). Separate fully adjusted logistic regression models were run within each subgroup to estimate the association between maternal ADHD and pregnancy outcomes. To assess whether the estimated associations differed across comorbidity levels, we performed an overall Wald chi‐squared test comparing log odds across groups. For outcomes showing significant differences in risk estimates, post hoc pairwise comparisons were conducted using Wald Z tests.

## 3 Results

### 3.1 Characteristics of Study Participants

A total of 977,266 women gave birth to a live singleton between January 1, 2006, and December 1, 2020 (*N* = 1,617,121 pregnancies). Of these 977,266 women, 12,553 (1.3%) were diagnosed with ADHD prior to pregnancy. These 12,553 women had a total of 17,434 pregnancies. Women with ADHD were younger at delivery than the controls (median age 25 versus 30 years) and were more likely to smoke during pregnancy. See Table 1 for background characteristics of the study population, by ADHD status.

**TABLE 1:** Background characteristics of study population of 1,617,121 pregnancies by ADHD status.

Maternal characteristics	ADHD	
	Yes *N* = 17,434 (%)	No *N* = 1,599,687 (%)
Age		
Median	27.0	30.0
15–24	6098 (35.0)	204,726 (12.8)
25–34	9272 (53.2)	1,039,311 (65.0)
35+	2064 (11.8)	355,650 (22.2)
Year of childbirth		
2006–2008	206 (1.2)	304,783 (19.1)
2009–2011	960 (5.5)	320,121 (20.0)
2012–2014	2716 (15.6)	322,256 (20.1)
2015–2017	5413 (31.0)	328,939 (20.6)
2018–2020	8139 (46.7)	323,588 (20.2)
Education		
≤ 9 years	5715 (32.8)	138,479 (8.7)
10–12 years	8395 (48.1)	550,577 (34.4)
≥ 13 years	3099 (17.8)	885,360 (55.3)
*Missing*	*225 (1.3)*	*25,271 (1.6)*
Comorbid mental disorders^a^		
Depression	6831 (39.2)	67,217 (4.2)
Anxiety	7828 (44.9)	77,198 (4.8)
Bipolar	1868 (10.7)	6914 (0.4)
Substance use disorders	4849 (27.8)	44,132 (2.8)
Cohabitation status		
Yes	13,003 (74.6)	1,428,575 (89.3)
No	3438 (19.7)	97,704 (6.1)
*Missing*	*993*	*73,408*
Maternal smoking		
Early in pregnancy	3816 (23.5)	82,368 (5.4)
Late in pregnancy	2955 (19.8)	60,559 (4.3)

^a^ Prior to pregnancy.

### 3.2 Main Results

#### 3.2.1 Gestational Age

Of the 17,434 pregnancies by women with ADHD, 1089 (6.6%) were preterm births (< 37 weeks), compared to 73,423 (4.9%) preterm births among women without an ADHD diagnosis. This corresponds to a crude OR of 1.33 (95% CI 1.25, 1.42). The association remained significant after adjusting for maternal age, year of childbirth, and maternal education (adjOR = 1.29, 95% CI 1.20, 1.38). However, the association was attenuated and became statistically nonsignificant after adjusting for comorbid mental disorders (adjOR = 1.06, 95% CI 0.99, 1.13) and smoking during pregnancy (adjOR = 1.06, 95% CI 0.97, 1.15). Women with ADHD were less likely to experience postterm birth (> 41 weeks) in both crude (OR = 0.77, 95% CI 0.72, 0.82) and adjusted models. However, after full adjustment, the association became nonsignificant (adjOR = 0.95, 95% CI 0.88, 1.03), see Table 2.

**TABLE 2:** Association between ADHD and pregnancy outcomes in women with versus without ADHD.

Pregnancy outcomes	*N* (%)	*N* (%)	Crude	Adjusted^a^	Adjusted^b^	Adjusted^c^
	ADHD: Yes (*N* = 17,434)	ADHD: No (*N* = 1,599,687)	OR (95% CI)	OR (95% CI)	OR (95% CI)	OR (95% CI)
Gestational age						
Normal (≥ 37 and ≤ 41 weeks)	15,459 (88.7)	1,420,122 (88.8)	**1**	**1**	**1**	**1**
Preterm birth (< 37 weeks)	1089 (6.6)	73,423 (4.9)	**1.33 (1.25, 1.42)**	**1.29 (1.20, 1.38)**	1.06 (0.99, 1.13)	1.06 (0.97, 1.15)
Postterm birth (> 41 weeks)	879 (5.4)	105,827 (6.9)	**0.77 (0.72, 0.82)**	**0.82 (0.76, 0.88)**	0.93 (0.87, 1.00)	0.95 (0.88, 1.03)
Intrauterine growth						
Normal for gestational age	16,249 (93.3)	1,506,021 (94.3)	**1**	**1**	**1**	**1**
Small for gestational age	438 (2.5)	36,429 (2.3)	**1.10 (1.00, 1.21)**	0.91 (0.83, 1.01)	**0.88 (0.80, 0.98)**	**0.80 (0.71, 0.90)**
Large for gestational age	721 (4.1)	55,205 (3.4)	**1.21 (1.12, 1.31)**	**1.29 (1.18, 1.40)**	**1.16 (1.06, 1.26)**	**1.16 (1.06, 1.26)**
Birth weight						
≤ 2500 g	737 (8.2)	49,768 (5.8)	**1.41 (1.30, 1.54)**	**1.19 (1.10, 1.30)**	1.01 (0.93, 1.11)	0.92 (0.82, 1.03)
2501–3500 g	1889 (47.0)	367,641 (43.3)	**1.11 (1.07, 1.15)**	0.99 (0.96, 1.03)	**0.94 (0.90, 0.97)**	**0.89 (0.85, 0.93)**
3501–4500 g	1785 (44.4)	419,363 (49.4)	**1**	**1**	**1**	**1**
> 4500 g	103 (2.6)	29,873 (3.5)	**0.96 (0.87, 1.05)**	1.04 (0.95, 1.14)	1.05 (0.96, 1.16)	1.08 (0.98, 1.20)
Obstetric outcomes						
Unassisted vaginal birth	13,201 (75.7)	1,225,180 (76.6)	**1**	**1**	**1**	**1**
Caesarean section (combined)	3263 (18.7)	258,086 (16.1)	**1.27 (1.19, 1.35)**	**1.36 (1.28, 1.45)**	**1.09 (1.03, 1.17)**	**1.09 (1.01, 1.16)**
Caesarean section (acute)	1734 (10.9)	142,658 (9.6)	**1.16 (1.10, 1.23)**	**1.22 (1.15, 1.29)**	1.05 (0.98, 1.11)	1.04 (0.97, 1.11)
Caesarean section (planned)	1529 (9.7)	115,428 (7.9)	**1.31 (1.21, 1.41)**	**1.49 (1.38, 1.62)**	**1.15 (1.06, 1.25)**	**1.16 (1.06, 1.26)**
Assisted vaginal delivery	927 (5.3)	111,086 (6.9)	**0.75 (0.70, 0.80)**	0.95 (0.88, 1.01)	0.96 (0.90, 1.03)	1.00 (0.93, 1.08)
Preeclampsia	1065 (6.1)	81,748 (5.1)	**1.24 (1.04, 1.47)**	**1.27 (1.06, 1.51)**	1.18 (0.98, 1.41)	1.21 (1.00, 1.47)
Gestational diabetes	685 (3.9)	44,441 (2.8)	**1.44 (1.16, 1.79)**	1.02 (0.82, 1.27)	1.00 (0.80, 1.26)	1.01 (0.80, 1.29)

*Note*: Bolded odds ratios indicate statistically significant associations at *p* < 0.05, i.e., the 95% confidence interval does not include 1.

^a^ Adjusted for maternal education, maternal age at childbirth, and year of childbirth.

^b^ Also adjusted for depression, anxiety, bipolar disorder, and SUDs prior to pregnancy.

^c^ Also adjusted for smoking during pregnancy.

#### 3.2.2 Intrauterine Growth

Women with ADHD had an increased risk of delivering LGA babies (crude OR = 1.21, 95% CI 1.12–1.31). This association remained statistically significant across all adjusted models, including full adjustment (adjOR = 1.16, 95% CI 1.06–1.26). Conversely, the risk of delivering SGA babies decreased in fully adjusted models (adjOR = 0.80, 95% CI 0.71–0.90), see Table 2.

#### 3.2.3 Birthweight

For low birthweight (≤ 2500 g), women with ADHD had a higher crude risk (OR = 1.41, 95% CI 1.30, 1.54), but the association was no longer statistically significant after full adjustment (adjOR = 0.92, 95% CI 0.82, 1.03). Similarly, women with ADHD were less likely to deliver babies weighing 2501–3500 g (adjOR = 0.89, 95% CI 0.85, 0.93) and more likely to deliver babies weighing > 4500 g, although this association was not significant in fully adjusted models (adjOR = 1.08, 95% CI 0.98, 1.20); see Table 2.

#### 3.2.4 Obstetric Outcomes

Women with ADHD had an increased likelihood of undergoing a planned caesarean section (crude OR = 1.31, 95% CI 1.21, 1.41). This association remained statistically significant after full adjustment (adjOR = 1.16, 95% CI 1.06, 1.26). Similarly, the risk of preeclampsia was elevated among women with ADHD in crude models (OR = 1.24, 95% CI 1.04, 1.47) but estimates approached the null after full adjustment (adjOR = 1.21, 95% CI 1.00, 1.47). For gestational diabetes, the association was statistically significant in crude models (OR = 1.44, 95% CI 1.16, 1.79) but attenuated after adjustment for covariates, including smoking during pregnancy (adjOR = 1.01, 95% CI 0.80, 1.29), see Table 2.

### 3.3 Sensitivity Analyses

#### 3.3.1 Broader Definition of ADHD

Analyses using a broader definition of ADHD, where women were defined as having ADHD if they had ever received a diagnosis or been dispensed medication for ADHD (*n* = 48,653 pregnancies), showed results largely consistent with the main findings. In both analyses, women with ADHD had an increased risk of delivering LGA babies and undergoing planned caesarean sections, while they were less likely to experience postterm birth, deliver SGA babies, or have an assisted vaginal delivery. However, some differences emerged in the fully adjusted models. The sensitivity analysis indicated statistically significant associations between ADHD and preterm birth (< 37 weeks; adjOR = 1.09, 95% CI 1.04, 1.15), as well as delivering a child weighing > 4500 g (adjOR = 1.13, 95% CI 1.06, 1.19). The sensitivity analysis also suggested a significant association between ADHD and acute caesarean section (adjOR = 1.09, 95% CI 1.04, 1.13). Conversely, the sensitivity analysis found a lower risk of low birthweight (≤ 2500 g; adjOR = 0.86, 95% CI 0.80, 0.92), whereas the main results showed a null association after full adjustment. For more detailed results, see Table S1.

#### 3.3.2 ADHD Medication During Pregnancy Excluded

After excluding women (*N* = 346) receiving a prescription of ADHD medication during pregnancy between 2006 and 2020, results were largely consistent with the main findings. Women with ADHD remained at an increased risk of undergoing planned caesarean sections. Similarly, the decreased risk of SGA babies and birthweights ≤ 2500 g or 2501–3500 g was also observed. Notably, this sensitivity analysis showed a statistically significant association between ADHD and preterm birth (adjOR = 1.20, 95% CI 1.04, 1.38), in contrast to the main results where this association was not statistically significant after full adjustment. The sensitivity analysis also suggested an increased risk of delivering babies weighing > 4500 g (adjOR = 1.18, 95% CI 1.11, 1.25), which approached the null in the main results. For more detailed results, see Table S2.

#### 3.3.3 First Pregnancy Only

Restricting the analyses to primipara women revealed results that were mostly aligned with the main findings. Women with ADHD were still at an increased risk of delivering LGA babies and undergoing planned caesarean sections. The decreased risk of SGA babies, birthweights between 2501 and 3500 g, and assisted vaginal deliveries was also evident. However, the association with preeclampsia (adjOR = 1.13, 95% CI 1.02, 1.26) was significant in this sensitivity analysis compared to the main results, where it approached the null after adjustment for comorbid mental disorders and smoking during pregnancy. For more detailed results, see Table S3.

#### 3.3.4 Mutually Exclusive ADHD Subgroups

To examine whether the definition of ADHD (diagnosis versus medication) influenced the association with pregnancy outcomes, we compared two mutually exclusive subgroups. Results showed largely similar adjusted ORs across groups, with no statistically significant differences based on ADHD definition. Although point estimates varied slightly for some outcomes, for example, SGA and planned caesarean section, Wald tests showed that none of these differences reached statistical significance (all *p* > 0.05). These findings suggest that associations were consistent regardless of how ADHD was defined. For full results, see Table S5.

#### 3.3.5 Comorbidity Burden

To examine whether the number of co‐occurring mental disorders influenced associations between ADHD and pregnancy outcomes, we stratified the sample into three groups: no comorbidity, one comorbidity, and two or more comorbidities. Fully adjusted logistic regression models were run within each group. An overall Wald test indicated significant differences in ADHD‐outcome associations across comorbidity levels for LGA births and planned caesarean sections. Post hoc pairwise comparisons revealed that the risk of LGA births was significantly higher in women with ADHD and no or one comorbidity, but not among those with two or more comorbid mental disorders. For planned caesarean sections, the risk was highest in women with no comorbidity, and significantly lower in those with one comorbid mental disorder. For more detailed results, see Table S6.

## 4 Discussion

In this population‐based study using nationwide Swedish population‐based registers, we examined the association between maternal ADHD and a range of pregnancy and neonatal outcomes. By linking data from over 1.6 million singleton pregnancies, we investigated outcomes such as preterm birth, birthweight, and caesarean sections, while accounting for key covariates, including comorbid mental disorders and smoking during pregnancy. Our main findings indicate that women diagnosed with ADHD prior to pregnancy were not at increased risk of preterm birth. Notably, after adjusting for comorbid mental disorders, the association between ADHD and preterm birth (< 37 weeks) became nonsignificant, suggesting that comorbidities may partly explain the initially observed risk in crude models. Women with ADHD had an increased likelihood of delivering a LGA baby and undergoing a planned caesarean section. In contrast, ADHD was associated with a lower likelihood of delivering a SGA baby or a baby with a birthweight between 2501 and 3500 g.

Our sensitivity analyses using a broader definition of ADHD identified a small increased risk of preterm birth (< 37 weeks) and acute caesarean section, which were not observed in the main analysis. However, the effect sizes observed in the sensitivity analysis were small, again suggesting that ADHD may not be a strong or clinically significant risk factor for preterm birth, over and above other established risk factors such as comorbid mental disorders and smoking during pregnancy.

In our population‐based sample, we identified only 3994 women (5471 pregnancies) with a sole diagnosis of ADHD. The majority of pregnancies involved women with ADHD who also had a comorbid mental disorder, engaged in smoking during pregnancy, or both. This is consistent with previous research indicating that ADHD often co‐occurs with other mental disorders [33] and risky lifestyle behaviors, such as smoking during pregnancy [13].

The present study extends previous research in two important ways. First, we defined ADHD based on clinical diagnoses and/or medication use prior to pregnancy, offering a more robust and comprehensive definition of ADHD compared to prior studies that often relied solely on medication use at a single timepoint [7]. Second, our findings highlight the importance of comorbid mental disorders in the association between ADHD and pregnancy outcomes, as adjusting for these disorders seemed to explain some of the associations between ADHD and pregnancy outcomes, such as preterm birth and low birthweight (≤ 2500 g).

Moreover, the present study revealed that women with ADHD were less likely to deliver a SGA baby or a baby with a birthweight of 2501–3500 g, which is consistent across both main and sensitivity analyses. At the same time, ADHD was associated with an increased likelihood of delivering a baby classified as being LGA or a baby weighing over 4500 g, suggesting that pregnancies affected by maternal ADHD may be associated with a tendency toward higher fetal growth. These findings warrant further research investigating potential underlying mechanisms, including the role of metabolic factors or genetic influences in women with ADHD.

It is widely acknowledged that ADHD often co‐occurs with other mental disorders such as depression and anxiety, as well as unhealthy behaviors like substance use. Furthermore, adolescents with ADHD are at an elevated risk of experiencing unplanned pregnancies [34] and becoming teenage parents [35]. The combination of being a teenage mother with the heightened risk of comorbid mental disorders and substance use could further increase the likelihood of pregnancy outcomes in young women diagnosed with ADHD. Therefore, adolescents and young adults with ADHD may need comprehensive healthcare support, including access to mental health services, to address potential challenges associated with ADHD. Interestingly, we did not observe a dose–response relationship between comorbid mental disorders and adverse pregnancy outcomes. The strongest associations between ADHD and outcomes such as LGA births and planned caesarean sections were found among women with no or only one comorbid mental disorder. One plausible explanation is that comorbid mental disorders may serve as a proxy for increased engagement with healthcare services. Women with multiple comorbidities may be more likely to receive ongoing mental health support and closer prenatal monitoring, which could attenuate the risks otherwise associated with maternal ADHD.

As the number of adults diagnosed with ADHD continues to rise [2], and with increasing use of ADHD medications among women of reproductive age [36], research on the potential risks of ADHD and its pharmacological treatment during pregnancy has expanded. To date, there are no established guidelines for evaluating the risk–benefit profile of ADHD medication use during pregnancy and the postpartum period [35]. Some researchers have advised against discontinuing ADHD medication for women with moderate to severe ADHD during pregnancy [37]. Importantly, this reflects ongoing clinical debate rather than a universal recommendation, and our study does not aim to evaluate or inform medication decisions during pregnancy. In our sample, very few women used ADHD medication during pregnancy, which underscores that medication exposure was uncommon. Rather than focusing solely on the potential risks of pharmacological treatment, our findings highlight the need to address the underlying disorder and associated comorbidities when considering pregnancy outcomes in women with ADHD.

### 4.1 Strengths and Limitations

The present study's strengths include the use of Swedish population‐based registers, which allowed for the inclusion of a large sample and the ability to adjust for several key covariates. The validity of the pregnancy outcomes we investigated in the current study is notably high, as indicated by previous research [22]. Despite this, it is important to acknowledge a few limitations. Some pregnancy outcomes (e.g., stillbirth, congenital malformations) were not included in the present study based on power constraints. Further, we acknowledge that the OR may approximate the risk ratio for rarer outcomes but diverge slightly for outcomes with higher prevalence, and this should be considered when interpreting our findings even though most of the studied outcomes remained rare (e.g., preterm birth: 6.6%; SGA: 2.5%; gestational diabetes: 3.9%; preeclampsia: 6.1%).

We adjusted for comorbid mental disorders (e.g., depression, anxiety, bipolar disorder, and SUD). It is worth noting that there could be other disorders that might influence the association between ADHD and pregnancy outcomes. However, the disorders used in the present study were included as they are commonly seen in women with ADHD and have been shown to be associated with adverse perinatal outcomes [10, 11, 28]. Also, it is worth noting that our ability to fully account for comorbid mental disorders may be limited by misclassification, as we only included diagnoses from specialist care. This means we are likely missing mental disorders diagnosed in primary care, as well as undiagnosed mental disorders in individuals who did not seek treatment. These factors may introduce residual confounding, potentially influencing the association between ADHD and pregnancy outcomes. Our analysis did not account for all possible confounders, and unmeasured confounding could still affect the results. Future large‐scale studies could include additional potential confounders to better understand the complex associations between ADHD and pregnancy outcomes.

A limitation in previous research on the association between ADHD treatment and pregnancy outcomes is the lack of accounting for ADHD severity [38]. This is noteworthy since ADHD severity is known to influence comorbidity rates and treatment strategies [39]. In the current study, information on ADHD subtype or severity was unavailable, though ADHD diagnoses identified from ICD criteria (by psychiatric specialists) generally capture more severe cases. The present study might underestimate smoking later in pregnancy, as this information will not be recorded for women giving birth to a preterm child before Weeks 30–32. Finally, the generalizability of our findings outside of the Swedish context may be limited due to differences in healthcare systems and ADHD diagnostic practices in other countries. A further limitation concerns the accuracy of ADHD classification. Although diagnoses in Swedish specialist care are typically made by licensed clinicians using ICD criteria, the quality and consistency of assessments may vary, particularly since adult ADHD diagnostics is a relatively recent development in Sweden [40]. Access to thorough evaluation is often limited by long waiting times and resource constraints. While national guidelines recommend that pharmacological treatment should only be initiated when psychosocial interventions have proven insufficient [41], and women with ADHD have been shown to exhibit high healthcare use and psychiatric service contacts [42], the possibility of misclassification remains. Such misclassification is likely nondifferential with respect to pregnancy outcomes and may attenuate the observed associations.

## 5 Conclusions

After adjustments for comorbid mental disorders and smoking during pregnancy, maternal ADHD was not associated with preterm birth. Women with ADHD had a slightly increased risk of delivering an LGA baby and undergoing a planned caesarean section.

## Author Contributions

**Anneli Andersson:** conceptualization, methodology, data curation, formal analysis, writing – original draft. **Miguel Garcia‐Argibay:** methodology, data curation, writing – review and editing. **Sofi Oskarsson:** methodology, data curation, writing – review and editing. **Jonas F. Ludvigsson:** writing – review and editing. **Paul Lichtenstein:** writing – review and editing. **Brian M. D'Onofrio:** writing – review and editing. **Catherine Tuvblad:** supervision, writing – review and editing. **Laura Ghirardi:** writing – review and editing. **Henrik Larsson:** conceptualization, writing – review and editing.

## Ethics Statement

All procedures involving human subjects were approved by the Swedish Ethical Review Authority (DNR: 2022–06204‐02). Since our study was based on population‐based registers, the requirement for informed consent was waived.

## Conflicts of Interest

Henrik Larsson reports receiving grants from Shire Pharmaceuticals; personal fees from and serving as a speaker for Medice, Shire/Takeda Pharmaceuticals, and Evolan Pharma AB; all outside the submitted work. Henrik Larsson is editor‐in‐chief of JCPP Advances. Dr. Ludvigsson has coordinated an unrelated study on behalf of the Swedish IBD quality register (SWIBREG). That study received funding from Janssen corporation. Dr. Ludvigsson has also received financial support from MSD developing a paper reviewing national healthcare registers in China. Dr. Ludvigsson is currently discussing potential research collaboration with Takeda. The other authors declare no conflicts of interest.

## Supporting information


**Data S1:** acps7003‐sup‐0001‐Tables.docx.

## Data Availability

Data cannot be shared publicly because of the Swedish Secrecy Act. Data from the Medical Birth Register, the Multigeneration Register, and the National Patient Register were used for this study and made available by ethical approval. Researchers may apply for access through the Swedish Research Ethics Boards (www.etikprovningsmyndigheten.se) and from the primary data owners Statistics Sweden (www.scb.se) and the National Board of Health and Welfare (www.socialstyrelsen.se), in accordance with Swedish law.
