# Supplementary material for: Pregnancy Outcomes in Women Diagnosed With Attention‐Deficit/Hyperactivity Disorder: A Population‐Based Register Study
Source: Acta Psychiatr Scand. 2025 Oct 1;153(1):34–43. doi: 10.1111/acps.70039 (PMC12668891; doi:10.1111/acps.70039)
Supplement: Supplementary file 1 — Data S1: acps7003‐sup‐0001‐Tables.docx. [file ACPS-153-34-s001.docx]

**Table S1.** Association between ADHD and pregnancy outcomes in women with versus without ADHD: Broader definition of ADHD.

|  | **N (%)** | **N (%)** | **Crude** | **Adjusted^a^** | **Adjusted^b^** | **Adjusted^c^** |
| --- | --- | --- | --- | --- | --- | --- |
| **Adverse pregnancy outcomes** | **ADHD: Yes**  **(N = 48,653)** | **ADHD: No**  **(N = 1,568,468)** | **OR (95% CI)** | **OR (95% CI)** | **OR (95% CI)** | **OR (95% CI)** |
| ***Gestational age*** |  |  |  |  |  |  |
| Normal (≥37 & ≤41 weeks) | 43,064 (88.5) | 1,392,517 (88.8) | **1** | **1** | **1** | **1** |
| Preterm birth (<37 weeks) | 2,956 (6.4) | 71,556 (4.9) | **1.31 (1.26, 1.37)** | **1.24 (1.19, 1.30)** | **1.12 (1.07, 1.17)** | **1.09 (1.04, 1.15)** |
| Post term birth (>41 weeks) | 2,619 (5.7) | 104,087 (6.9) | **0.82 (0.79, 0.85)** | **0.82 (0.79, 0.86)** | **0.88 (0.84, 0.92)** | **0.91 (0.87, 0.95)** |
| ***Intrauterine growth*** |  |  |  |  |  |  |
| Normal for gestational age | 45,316 (93.3) | 1,476,954 (94.3) | **1** | **1** | **1** | **1** |
| Small for gestational age | 1,117 (2.3) | 35,750 (2.3) | 1.01 (0.95, 1.08) | **0.89 (0.84, 0.95)** | **0.87 (0.82, 0.93)** | **0.77 (0.72, 0.83)** |
| Large for gestational age | 2,132 (4.4) | 53,794 (3.4) | **1.27 (1.21, 1.34)** | **1.35 (1.28, 1.42)** | **1.29 (1.22, 1.36)** | **1.36 (1.28, 1.43)** |
| ***Birth weight*** |  |  |  |  |  |  |
| ≤2500 g | 1,828 (7.3) | 48,677 (5.8) | **1.26 (1.19, 1.32)** | **1.10 (1.05, 1.17)** | 1.01 (0.95, 1.06) | **0.86 (0.80, 0.92)** |
| 2501-3500 g | 21,769 (44.8) | 679,249 (43.3) | **1.08 (1.06, 1.11)** | 0.99 (0.97, 1.01) | **0.96 (0.94, 0.98)** | **0.90 (0.88, 0.92)** |
| 3501-4500 g | 23,325 (48.0) | 785,481 (50.1) | **1** | **1** | **1** | **1** |
| >4500 g | 1,659 (3.4) | 53,445 (3.4) | 1.04 (0.99, 1.10) | **1.08 (1.02, 1.14)** | **1.09 (1.03, 1.15)** | **1.13 (1.06, 1.19)** |
| ***Obstetric outcomes*** |  |  |  |  |  |  |
| Unassisted vaginal birth | 35,648 (73.3) | 1,165,822 (74.3) | **1** | **1** | **1** | **1** |
| Caesarean section (combined) | 9,150 (18.8) | 252,199 (16.1) | **1.23 (1.18, 1.28)** | **1.37 (1.32, 1.43)** | **1.22 (1.17, 1.27)** | **1.22 (1.17, 1.27)** |
| Caesarean section (acute) | 4,687 (10.6) | 139,705 (9.6) | **1.13 (1.08, 1.17)** | **1.19 (1.14, 1.23)** | **1.09 (1.05, 1.14)** | **1.09 (1.04, 1.13)** |
| Caesarean section (planned) | 4,463 (10.1) | 112,494 (7.9) | **1.32 (1.25, 1.38)** | **1.58 (1.50, 1.66)** | **1.38 (1.31, 1.46)** | **1.39 (1.32, 1.46)** |
| Assisted vaginal delivery | 2,849 (5.9) | 109,164 (6.9) | **0.83 (0.80, 0.86)** | **0.88 (0.84, 0.91)** | **0.88 (0.85, 0.92)** | **0.91 (0.83, 0.99)** |
| Preeclampsia | 2,888 (5.9) | 79,925 (5.1) | 1.22 (1.09, 1.36) | 1.22 (1.09, 1.37) | 1.17 (1.04, 1.32) | **1.20 (1.06, 1.35)** |
| Gestational diabetes | 1,487 (3.1) | 43,639 (2.8) | 1.09 (0.94, 1.27) | 0.98 (0.84, 1.15) | 0.98 (0.83, 1.15) | 0.99 (0.84, 1.16) |

Note. ^a^Adjusted for highest achieved maternal education at childbirth; maternal age at childbirth; and infant year of birth. ^b^Also adjusted for depression, anxiety, bipolar disorder, and SUDs prior to pregnancy. ^c^Also adjusted for smoking during pregnancy.

**Table S2.** Association between ADHD and pregnancy outcomes in women with versus without ADHD: Medication during pregnancy excluded.

|  | **N (%)** | **N (%)** | **Crude** | **Adjusted^a^** | **Adjusted^b^** | **Adjusted^c^** |
| --- | --- | --- | --- | --- | --- | --- |
| **Adverse pregnancy outcomes** | **ADHD: Yes**  **(N = 48,307)** | **ADHD: No**  **(N = 1,568,468)** | **OR (95% CI)** | **OR (95% CI)** | **OR (95% CI)** | **OR (95% CI)** |
| ***Gestational age*** |  |  |  |  |  |  |
| Normal (≥37 & ≤41 weeks) | 3,390 (85.9) | 747,755 (88.1) | **1** | **1** | **1** | **1** |
| Preterm birth (<37 weeks) | 327 (8.3) | 44,973 (5.3) | **1.58 (1.40, 1.78)** | **1.48 (1.31, 1.67)** | **1.25 (1.11, 1.42)** | **1.20 (1.04, 1.38)** |
| Post term birth (>41 weeks) | 228 (5.8) | 56,255 (6.6) | 0.90 (0.78, 1.03) | 0.94 (0.82, 1.08) | 1.03 (0.89, 1.19) | 1.04 (0.90, 1.21) |
| ***Intrauterine growth*** |  |  |  |  |  |  |
| Normal for gestational age | 3,662 (92.8) | 787,847 (92.8) | **1** | **1** | **1** | **1** |
| Small for gestational age | 97 (2.5) | 18,698 (2.2) | 1.12 (0.91, 1.37) | 0.84 (0.68, 1.04) | 0.81 (0.65, 1.00) | **0.69 (0.54, 0.88)** |
| Large for gestational age | 129 (3.3) | 29,203 (4.4) | 0.96 (0.79, 1.15) | 1.03 (0.85, 1.25) | 0.93 (0.77, 1.13) | 1.00 (0.81, 1.22) |
| ***Birth weight*** |  |  |  |  |  |  |
| ≤2500 g | 1,797 (7.2) | 48,677 (5.8) | **1.24 (1.18, 1.31)** | **1.09 (1.03, 1.15)** | 1.00 (0.94, 1.05) | **0.86 (0.80, 0.92)** |
| 2501-3500 g | 21,587 (44.7) | 679,249 (43.3) | **1.06 (1.04, 1.08)** | 0.98 (0.96, 1.00) | **0.95 (0.93, 0.97)** | **0.90 (0.88, 0.92)** |
| 3501-4500 g | 23,204 (48.1) | 785,481 (50.1) | **1** | **1** | **1** | **1** |
| >4500 g | 1,647 (3.4) | 53,445 (3.4) | 1.00 (0.95, 1.06) | **1.07 (1.02, 1.13)** | **1.00 (1.04, 1.16)** | **1.18 (1.11, 1.25)** |
| ***Obstetric outcomes*** |  |  |  |  |  |  |
| Unassisted vaginal birth | 2,888 (73.2) | 638,669 (75.2) | **1** | **1** | **1** | **1** |
| Caesarean section (combined) | 755 (20.2) | 137,761 (17.2) | **1.24 (1.12, 1.38)** | **1.45 (1.30, 1.61)** | **1.20 (1.08, 1.34)** | **1.21 (1.08, 1.35)** |
| Caesarean section (acute) | 407 (10.3) | 77,168 (9.1) | **1.17 (1.05, 1.31)** | **1.26 (1.12, 1.41)** | 1.09 (0.97, 1.23) | 1.12 (0.99, 1.27) |
| Caesarean section (planned) | 348 (8.8) | 60,593 (7.1) | **1.26 (1.11, 1.44)** | **1.68 (1.47, 1.91)** | **1.33 (1.16, 1.52)** | **1.32 (1.14, 1.52)** |
| Assisted vaginal delivery | 275 (6.6) | 67,134 (7.5) | **0.87 (0.77, 0.99)** | 0.99 (0.87, 1.12) | 1.01 (0.89, 1.15) | 1.07 (0.93, 1.22) |
| Preeclampsia | 212 (5.4) | 42,832 (5.0) | 1.08 (0.78, 1.48) | 1.14 (0.83, 1.58) | 1.09 (0.78, 1.52) | 1.10 (0.78, 1.56) |
| Gestational diabetes | 58 (1.5) | 13,986 (1.6) | 0.90 (0.50, 1.61) | 0.87 (0.48, 1.59) | 0.82 (0.44, 1.51) | 0.76 (0.39, 1.46) |

Note. ^a^Adjusted for highest achieved maternal education at childbirth; maternal age at childbirth; and infant year of birth. ^b^Also adjusted for depression, anxiety, bipolar disorder, and SUDs prior to pregnancy. ^c^Also adjusted for smoking during pregnancy.

**Table S3.** Association between ADHD and pregnancy outcomes in women with versus without ADHD: First pregnancy only.

|  | **N (%)** | **N (%)** | **Crude** | **Adjusted^a^** | **Adjusted^b^** | **Adjusted^c^** |
| --- | --- | --- | --- | --- | --- | --- |
| **Adverse pregnancy outcomes** | **ADHD: Yes**  **(N = 9,082)** | **ADHD: No**  **(N = 698,612)** | **OR (95% CI)** | **OR (95% CI)** | **OR (95% CI)** | **OR (95% CI)** |
| ***Gestational age*** |  |  |  |  |  |  |
| Normal (≥37 & ≤41 weeks) | 7,943 (87.5) | 600,085 (85.9) | **1** | **1** | **1** | **1** |
| Preterm birth (<37 weeks) | 554 (6.5) | 39,177 (6.1) | 1.07 (0.98, 1.17) | 1.08 (0.99, 1.18) | 0.97 (0.89, 1.07) | 0.98 (0.87, 1.10) |
| Post term birth (>41 weeks) | 582 (6.8) | 59,230 (9.0) | **0.74 (0.68, 0.81)** | **0.82 (0.75, 0.89)** | **0.91 (0.83, 0.99)** | 0.92 (0.83, 1.01) |
| ***Intrauterine growth*** |  |  |  |  |  |  |
| Normal for gestational age | 8,527 (93.9) | 661,108 (94.6) | **1** | **1** | **1** | **1** |
| Small for gestational age | 294 (3.2) | 23,352 (3.3) | 0.97 (0.86, 1.09) | **0.80 (0.71, 0.91)** | **0.80 (0.71, 0.91)** | **0.75 (0.66, 0.87)** |
| Large for gestational age | 252 (2.8) | 13,176 (1.9) | **1.48 (1.31, 1.68)** | **1.39 (1.23, 1.59)** | **1.21 (1.06, 1.38)** | **1.28 (1.11, 1.49)** |
| ***Birth weight*** |  |  |  |  |  |  |
| ≤2500 g | 429 (9.6) | 28,990 (8.6) | **1.13 (1.02, 1.25)** | 1.00 (0.90, 1.11) | 0.93 (0.84, 1.03) | 0.88 (0.77, 1.01) |
| 2501-3500 g | 4,402 (48.5) | 343,701 (49.3) | 0.97 (0.93, 1.01) | **0.91 (0.88, 0.95)** | **0.91 (0.87, 0.95)** | **0.86 (0.82, 0.91)** |
| 3501-4500 g | 4,042 (44.5) | 309,394 (44.3) | **1** | **1** | **1** | **1** |
| >4500 g | 203 (2.2) | 15,694 (2.2) | 0.99 (0.86, 1.14) | 1.04 (0.91, 1.20) | 1.06 (0.91, 1.22) | 1.10 (0.94, 1.29) |
| ***Obstetric outcomes*** |  |  |  |  |  |  |
| Unassisted vaginal birth | 8,301 (91.4) | 609,780 (87.3) | **1** | **1** | **1** | **1** |
| Caesarean section (combined) | 1,743 (19.2) | 123,918 (17.7) | **1.10 (1.05, 1.16)** | **1.24 (1.18, 1.31)** | **1.05 (1.00, 1.11)** | 1.06 (0.99, 1.12) |
| Caesarean section (acute) | 1,153 (13.6) | 89,392 (13.5) | 1.01 (0.95, 1.08) | **1.10 (1.03, 1.17)** | 0.99 (0.93, 1.06) | 0.99 (0.92, 1.07) |
| Caesarean section (planned) | 590 (7.4) | 34,526 (5.7) | **1.34 (1.23, 1.46)** | **1.64 (1.51, 1.79)** | **1.20 (1.10, 1.32)** | **1.21 (1.10, 1.34)** |
| Assisted vaginal delivery | 780 (8.6) | 88,479 (12.7) | **0.65 (0.60, 0.70)** | **0.84 (0.78, 0.90)** | **0.87 (0.81, 0.94)** | **0.91 (0.83, 0.98)** |
| Preeclampsia | 513 (5.6) | 36,177 (5.2) | 1.10 (1.00, 1.20) | **1.10 (1.01, 1.21)** | 1.07 (0.98, 1.18) | **1.13 (1.02, 1.26)** |
| Gestational diabetes | 299 (3.3) | 16,606 (2.4) | **1.40 (1.24, 1.57)** | 1.01 (0.90, 1.14) | 0.99 (0.88, 1.12) | 0.99 (0.87, 1.14) |

Note. ^a^Adjusted for highest achieved maternal education at childbirth; maternal age at childbirth; and infant year of birth. ^b^Also adjusted for depression, anxiety, bipolar disorder, and SUDs prior to pregnancy. ^c^Also adjusted for smoking during pregnancy.

**Table S4.** ICD-codes included to define the presence of comorbid psychiatric disorders prior to pregnancy.

| **Psychiatric disorder** | **ICD-8** | **ICD-9** | **ICD-10** |
| --- | --- | --- | --- |
| **Depression** |  | 296B, 298A, 296W, 311X, 300E, 300F, 309A, 309B | F381, F488, F4321, F32, F33, F34 |
| **Anxiety** |  | 300A, 300C, 300D | F40, F41, F42 |
| **Bipolar disorder** | 296, 298.1 | 296.0, 296.1, 296.4, 296.5, 296.6, 296.7, 298B, 296.80, 296.81, 296.89 | F34.0, F30, F31 |
| **Substance use disorders** | 303, 304 | 303, 304, 305 | F10-F19 |

Note. Based on the Swedish version of the International Classification of Diseases (ICD) version 8, 9 and 10 codes for
the identification of diagnosis.

**Table S5.** Comparison of adjusted OR for pregnancy outcomes between women with ADHD diagnosis only and ADHD medication only.

| **Pregnancy outcomes** | **ADHD diagnosis only (N = 4,290)**  **Adjusted^c^ OR (95% CI)** | **ADHD medication only (N = 4,410)**  **Adjusted^c^ OR (95% CI)** | **P-value** |
| --- | --- | --- | --- |
| ***Gestational age*** |  |  |  |
| Normal (≥37 & ≤41 weeks) | **1** | **1** |  |
| Preterm birth (<37 weeks) | 1.08 (0.91, 1.27) | 1.08 (0.92, 1.27) | 0.957 |
| Post term birth (>41 weeks) | 0.91 (0.78, 1.06) | 1.10 (0.95, 1.26) | 0.071 |
| ***Intrauterine growth*** |  |  |  |
| Normal for gestational age | **1** | **1** |  |
| Small for gestational age | 0.91 (0.73, 1.13) | **0.75 (0.59, 0.95)** | 0.238 |
| Large for gestational age | **1.28 (1.07, 1.53)** | 1.06 (0.88, 1.28) | 0.078 |
| ***Birth weight*** |  |  |  |
| ≤2500 g | 1.01 (0.83, 1.24) | 0.98 (0.80, 1.20) | 0.746 |
| 2501-3500 g | **0.92 (0.85, 0.99)** | 0.94 (0.87, 1.01) | 0.509 |
| 3501-4500 g | **1** | **1** |  |
| >4500 g | 1.01 (0.83, 1.24) | 1.10 (0.90, 1.33) | 0.647 |
| ***Obstetric outcomes*** |  |  |  |
| Unassisted vaginal birth | **1** | **1** |  |
| Caesarean section (combined) | 1.09 (0.96, 1.25) | **1.22 (1.08, 1.39)** | 0.133 |
| Caesarean section (acute) | 1.08 (0.95, 1.23) | 1.04 (0.92, 1.17) | 0.521 |
| Caesarean section (planned) | 1.16 (0.98, 1.36) | **1.37 (1.20, 1.56)** | 0.090 |
| Assisted vaginal delivery | 0.92 (0.80, 1.08) | 1.01 (0.88, 1.16) | 0.329 |
| Preeclampsia | 1.15 (0.80, 1.66) | 1.22 (0.86, 1.73) | 0.799 |
| Gestational diabetes | 0.99 (0.63, 1.56) | 0.92 (0.58, 1.45) | 0.751 |

**Note.** Reported OR and 95% CI are derived from the fully adjusted model, model c, which includes adjustments for maternal education,
age at childbirth, year of childbirth, history of depression, anxiety, bipolar disorder, SUDs, and smoking during pregnancy.

**Table S6**. Pregnancy outcomes by number of comorbid mental disorders in women with ADHD: Adjusted^c^ ORs with 95% CIs and p-values from Wald tests comparing comorbidity groups.

|  | **0 comorbid mental disorders** | **1 comorbid mental disorder** | **2+ comorbid mental disorders** | **P-value** |
| --- | --- | --- | --- | --- |
| **Pregnancy outcomes** | **Adjusted^c^ OR (95% CI)** | **Adjusted^c^ OR (95% CI)** | **Adjusted^c^ OR (95% CI)** |  |
| ***Gestational age*** |  |  |  |  |
| Normal (≥37 & ≤41 weeks) | **1** | **1** | **1** |  |
| Preterm birth (<37 weeks) | 1.09 (0.93, 1.27) | 1.04 (0.90, 1.21) | 1.05 (0.91, 1.21) | 0.908 |
| Post term birth (>41 weeks) | 0.99 (0.88, 1.12) | 0.90 (0.78, 1.04) | 1.14 (0.99, 1.32) | 0.075 |
| ***Intrauterine growth*** |  |  |  |  |
| Normal for gestational age | **1** | **1** | **1** |  |
| Small for gestational age | **0.70 (0.57, 0.87)** | 0.94 (0.76, 1.15) | 0.90 (0.73, 1.10) | 0.117 |
| Large for gestational age | **1.46 (1.24, 1.71)** | **1.34 (1.14, 1.56)** | 0.94 (0.80, 1.12) | **0.001*** |
| ***Birth weight*** |  |  |  |  |
| ≤2500 g | 0.87 (0.72, 1.06) | 1.11 (0.92, 1.33) | 0.97 (0.81, 1.17) | 0.218 |
| 2501-3500 g | **0.88 (0.82, 0.94)** | **0.92 (0.85, 0.98)** | **0.93 (0.86, 0.99)** | 0.584 |
| 3501-4500 g | **1** | **1** | **1** |  |
| >4500 g | **1.19 (1.01, 1.41)** | 1.09 (0.91, 1.31) | 1.00 (0.83, 1.21) | 0.401 |
| ***Obstetric outcomes*** |  |  |  |  |
| Unassisted vaginal birth | **1** | **1** | **1** |  |
| Caesarean section (combined) | **1.17 (1.04, 1.32)** | 1.08 (0.97, 1.21) | **1.15 (1.04, 1.28)** | 0.611 |
| Caesarean section (acute) | 1.03 (0.92, 1.15) | 1.09 (0.98, 1.22) | 1.09 (0.98, 1.22) | 0.685 |
| Caesarean section (planned) | **1.36 (1.20, 1.55)** | 1.07 (0.94, 1.22) | **1.15 (1.03, 1.30)** | **0.029*** |
| Assisted vaginal delivery | 0.93 (0.82, 1.06) | 1.08 (0.95, 1.23) | 1.02 (0.89, 1.16) | 0.265 |
| Preeclampsia | **1.58 (1.16, 2.14)** | 1.05 (0.77, 1.43) | 0.97 (0.72, 1.29) | 0.055 |
| Gestational diabetes | 1.01 (0.66, 1.52) | 1.17 (0.81, 1.69) | 1.02 (0.73, 1.44) | 0.828 |

Note. Reported OR and 95% CI are derived from the fully adjusted model, model c, which includes adjustments for maternal education, age at childbirth, year of childbirth, and smoking during pregnancy. *Pairwise p-values for outcomes with significant overall differences: Large for gestational age (0 vs 1 = 0.454, 0 vs 2+ = 0.000, 1 vs 2+ = 0.003); Planned caesarean section (0 vs 1 = 0.010, 0 vs 2+ = 0.057, 1 vs 2+ = 0.419).
